# Protein structure and selection pressure in plants: using mutation to understand the functional importance of protein structure

**DOI:** 10.1186/s12864-026-12674-2

**Published:** 2026-02-24

**Authors:** Evan Long, Grey Monroe

**Affiliations:** 1 United States Department of Agriculture - Agricultural Research Service, Northwest Irrigation and Soils Research Laboratory, Kimberly, ID 83341 USA; 2 Department of Plant Sciences, University of California Davis, Davis, CA 95616 USA

**Keywords:** Protein Structure, Protein Folding, Mutation, Selection Pressure

## Abstract

Recent advances in protein structure prediction have opened new avenues for understanding the potential impact of genetic mutations and how they might affect protein structure. In this study, we analyzed the distribution and structural characteristics of mutations in four plant species: Arabidopsis, rice, sugar beet, and cassava. We integrated population genotype datasets with protein structure predictions to map mutation positions to their corresponding gene protein products and structural features. Our analysis reveals that high-effect mutations are more likely to occur in unstructured, disordered regions of proteins rather than in well-folded, conserved regions. This finding suggests that natural selection exerts greater pressure to conserve sequence integrity in folded regions, which are crucial for protein function. Conversely, disordered regions may tolerate higher variability exhibiting a higher frequency of impactful mutations. By providing a comprehensive overview of mutation distribution in relation to protein structure, this study enhances our understanding of the evolutionary pressures shaping plant proteomes. The insights gained from this research could inform future studies on protein function, evolutionary biology, and plant improvement strategies.

**Supplementary Information:**

The online version contains supplementary material available at 10.1186/s12864-026-12674-2.

## Introduction

Ancestral and de novo mutations are responsible for the genetic diversity observed in various plant species. Understanding the functional impact of these mutations and how to incorporate those which are beneficial into modern crop varieties remains the major focus of crop breeders and geneticists. Standard breeding practices take many years to achieve the introgression of specific genomic loci. This process is even more challenging in plants with long reproductive periods, such as tree crops, or in highly heterozygous crops that suffer from inbreeding depression, such as alfalfa, potato, and cassava. Genome engineering, specifically gene editing with tools such as CRISPR-Cas9, has been presented as a possible method to address these challenges [1]. While many genome mapping studies are devoted to finding putative targets for locus introgression or genome editing, effectively identifying these targets, incorporating them, and producing the desired outcomes remain very difficult.

One large axis of functional variation is the sequence and structure of plant proteins. Recent advances in 3D model prediction of protein structure have enabled the accurate reconstruction of protein structure directly from protein sequence. The recent success of the tool AlphaFold [2], along with competitors such as ESM-fold [3], have enabled highly accurate protein structure prediction without requiring extensive X-ray crystallography experiments. It is still yet unknown how well these in silico reconstruction of protein structures can be used to estimate effects of single point mutations [4], however, some studies have shown high correlations between AlphaFold predicted importance and disease pathogenicity of non-synonymous mutations [5]. A protein’s 3D structure ultimately defines its function, allowing for more informed assessments of what mutations may define or disrupt overall protein activity.

**Fig. 1:**
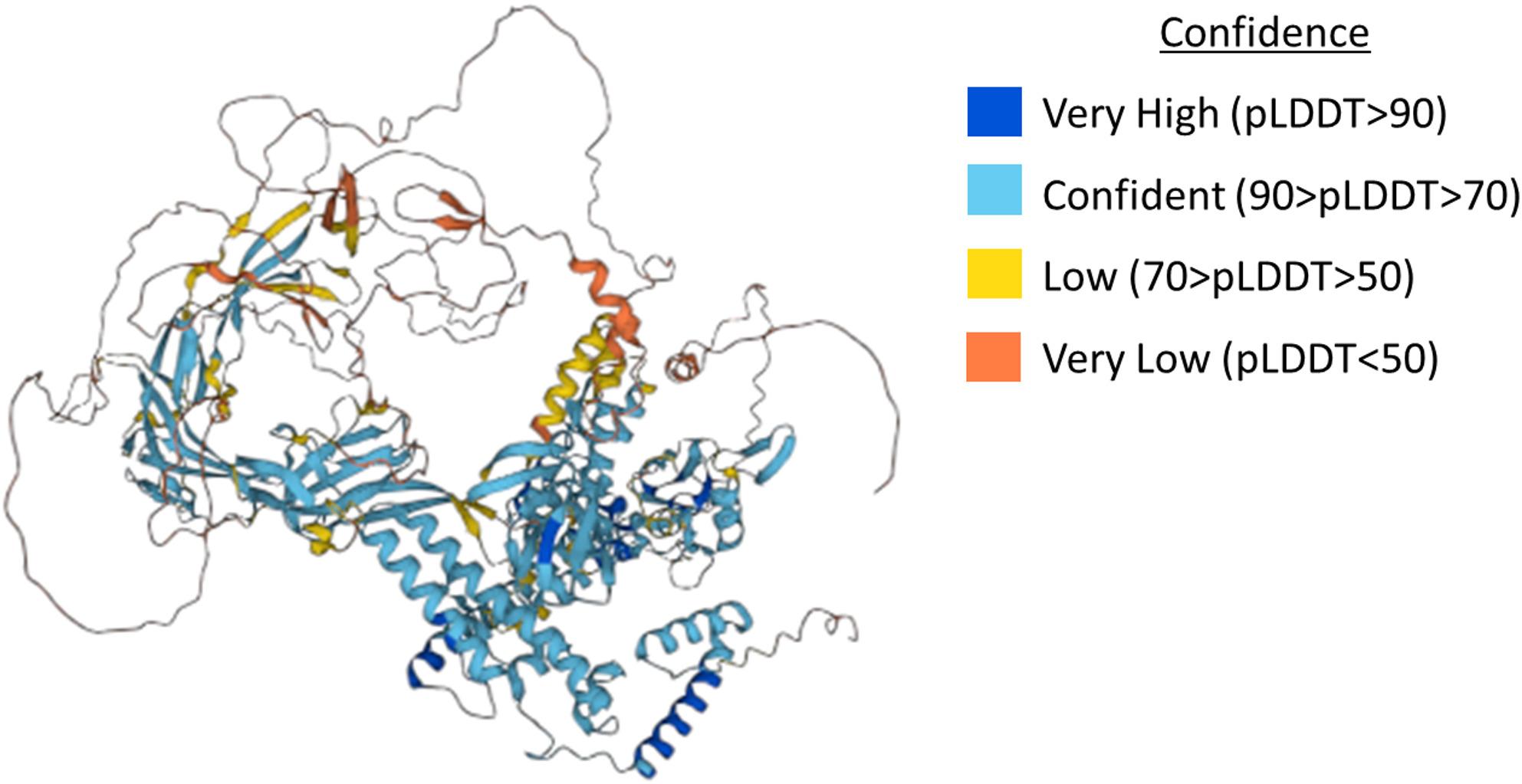
Example protein 3D structure. Protein structure of DNA-directed RNA polymerase subunit beta from cassava. Protein structure and image taken from https://alphafold.ebi.ac.uk/entry/B1NWE0

By analyzing the protein structures across an entire genome, we can identify formative characteristics that can help determine functional important regions of a protein. Two simple measurements resulting from protein structure prediction models that can be used to evaluate amino acid residues in folded protein structures are:

1. Predicted Local Distance Difference Test (pLDDT) score ranges between 0 and 100 and corresponds to the confidence at which the model gives the residue prediction (Fig. 1).
2. Relative accessible surface area (rASA) value ranging from 0-1 which corresponds to the percentage of the amino acid that is accessible or uncovered. This score in general can be used to assess if a residue is part of a folded structure (low rASA) or is part of a disordered region (high rASA).

With the emergence of these protein 3D structure resources, it’s necessary to evaluate how they can increase our understanding of mutation effects. One mutation class of special interest is loss-of-function (LoF) mutations because they are potentially high value targets that can be effectively targeted by genome editing [6]. These mutations are also commonly referred to as knockout mutations and are generally attributed to mutations that cause large disruptions to protein coding sequence such as frameshift, start-lost, stop-gain, and large insertions and deletions. The consequence of these mutations is generally assumed to be an inactive gene product. While LoF mutations are often thought of as being deleterious, many have been shown to be abundant in natural populations and can have impactful beneficial and adaptive effects in many crop species [7]. Additionally, LoF mutations are among the types of mutations that are most efficiently induced through gene editing, because among the simplest targeted gene editing methods are those that result in double stranded breaks resolved through non-homologous end joining (NHEJ) repair, often resulting in a knockout mutation [8].

Population genetic measures of selection pressure offer an independent validation of functional impacts of mutation. Large population genotype databases offer deep measurement for selection pressures across plant genomes. In this study we use these measurements of selection in rice [9], *Arabidopsis thaliana* [10], Cassava [11], and sugar beet [12], to evaluate the ability of predicted protein structure to explain mutational impact. We also introduce a pipeline to enable evaluations of relationships between protein structure and mutation effects for any species, given a population genotype dataset and protein structures for its annotated genes.

## Methods

### Protein structures

Protein structures were obtained for four plant species: rice (Oryza sativa), arabidopsis (*Arabidopsis thaliana)*, cassava (*Manihot esculenta*), and sugar beet (Beta vulgaris). To obtain protein structures for using a unified methodology, we used ESM-fold (model esm2_t33_650M_UR50D) [3] to fold primary transcripts for all protein coding genes for each species. While ESM-fold’s reported accuracy is below that of AlphaFold, its speed allows for widespread implementation across species of interest. The pLLDT folding confidence scores are reported in the protein data bank (PDB), and was averaged across residues corresponding to each amino acid. The available surface area for each amino acid was calculated using dssp [13] (commands available in https://github.com/em255/PopulationPDBStats), and divided by a theoretical maximum surface area [14] to produce the rASA for each amino acid.

### Population genetic datasets

Genome wide genotype files were obtained for each species in variant call format (VCF, Table 1). Arabidopsis genotypes were obtained from the 1000 genomes project (*1001genomes.org/data/GMI-MPI/releases/v3.1/*) [10]. Rice genotypes were obtained from the rice 3000 genomes project (*snp-seek.irri.org/_download.zul*) [9] including both SNPS and structural variants. Rice genotypes were filtered down to only *Oryza sativa subsp. Indica* in order to minimize impact of population structure similar to previous studies in rice [15, 16]. Sugar beet genotypes were downloaded from a study including a diversity survey of 600 + individuals [12]. Cassava genotypes were derived from a diverse set of Colombian landraces as part of a climate adaptation study [11].

**Table 1:** Plant Genotype Datasets

Title	Species	# Samples	# Variants	Source
Arabidopsis	*Arabidopsis thaliana*	1135	12.8 M	Alonso-Blanco et al. [10]
Rice	*Oryza Sativa subsp. Indica*	1425	31.9 M	Wang et al. [9]
Cassava	*Manihot esculenta*	420	71.5 M	Zhao et al. [11]
Sugar Beet	*Beta vulgaris*	667	8.1 M	Felkel et al. [12]

The genotype VCF files were procured from previously published studies. The number of different genotypes and variants in each dataset are presented

We used the tool snpEff (V5.2) [17] to measure the predicted effect of mutations on protein products. SnpEff uses the reference genome with its associated annotation (gff file) to classify the effect of all mutations in a genotype file (vcf). Mutations within gene coding regions of the genome are classified into three grades of effects low, moderate, and high, which largely correspond to variants that result in no change to amino acid sequence, single changes to amino acid sequence, and disruptive changes such as frameshift mutations or an early stop-codon, respectively. Each of the four plant species were annotated with snpEFF to measure mutations using default parameters according to published guides (https://pcingola.github.io/SnpEff/snpeff/build_db/). All transcripts were measured; however canonical or primary transcripts were used for the remainder of this study. To verify the integrity of ESM protein structure predictions we also compared protein characteristics with IUPreD2A [18] protein disorder and evolutionary conservation (https://plantregmap.gao-lab.org/download.php#alignment-conservation) in Arabidopsis.

### Mutations across protein structures

Mutations, along with their impact and population allele frequency, were extracted from these annotated files and compared to their corresponding positions along protein structures. We developed a toolkit (https://github.com/em255/PopulationPDBStats*)* to match mutational positions with protein positions to extract feature characteristics including pLDDT and rASA and compare to corresponding mutation characteristics. For the protein structure analysis without regard to mutations, 1 M amino acid positions were sampled from each species. Each species had 0.5 M-1.5 M nucleotide variant positions (single point mutations and insertions/deletions) mapped to protein structures used in this study.

In addition to snpEFF mutation effects, we also used classification of mutations by dissecting synonymous, nonsynonymous mutations, and high effect mutations. Nonsynonymous mutations were divided into two classes: Those resulting in a conserved/common codon for the respective amino acid, and those resulting in a non-conserved/uncommon codon for the respective amino acid, with classifications of conservation derived from codon frequencies for each amino acid in each species. Nonsynonymous mutations were classified as whether they resulted in an amino acid of a different functional class (radical) or the same functional class (conservative) relative to the original amino acid. Finally high effect mutations were analyzed as whether they resulted from a frameshift mutation, stop-gained mutation, or a start-lost mutation.

### Statistical analysis

We assessed whether rASA was significantly associated with MAF while controlling for amino acid identity and proportional position within the protein. To account for non-independence among observations from the same transcript, we fit a linear mixed-effects model using the lmer function in the lme4 package in R. The full model included rASA, AminoAcid (categorical), and AA_ProportionalPosition (numeric) as fixed effects, and transcript as a random intercept. We compared the full model to a reduced model without rASA using a likelihood ratio test (LRT) with maximum likelihood estimation (REML = FALSE) to determine the significance of rASA. Additonally, we performed pairwise Wilcoxon ratio tests between snpEFF effect classes and rASA for each species in R using the *pairwise.wilcox.test* function.

## Results

**Fig. 2:**
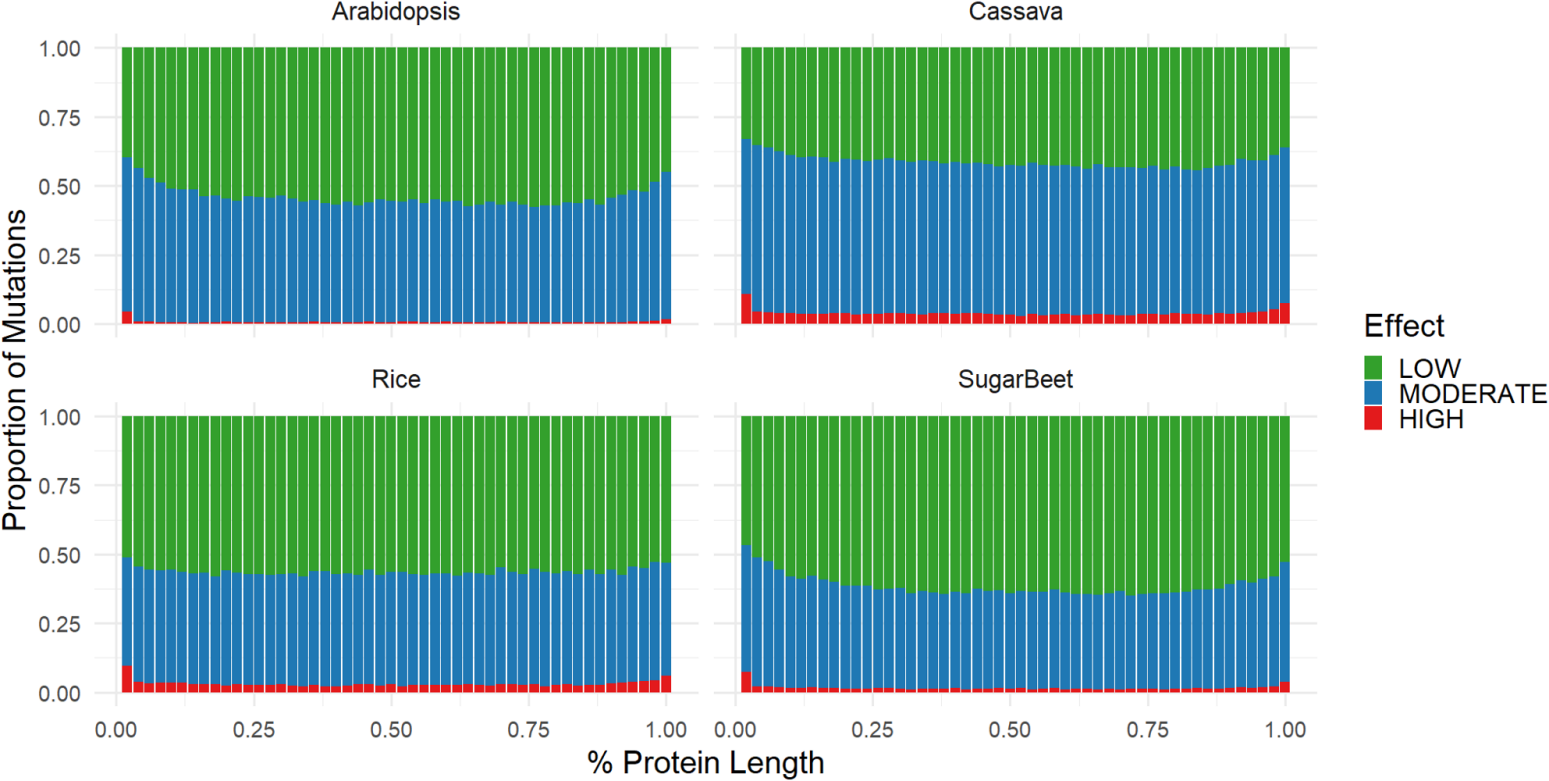
Relationship Between Amino Acid Positions and snpEFF Mutation Classes (Rare Varients Excluded). The proportion of mutations that fall into each snpEFF mutation class is plotted along the relative position of each amino acid of every protein coding gene in each plant genome. Mutation effects are classified as low (synonymous), moderate (nonsynonymous or in-frame small indels), and high (frameshift, splice site variants, start-codon loss, etc). Rare mutations with a minor allele frequency less than 5% were excluded

**Fig. 3:**
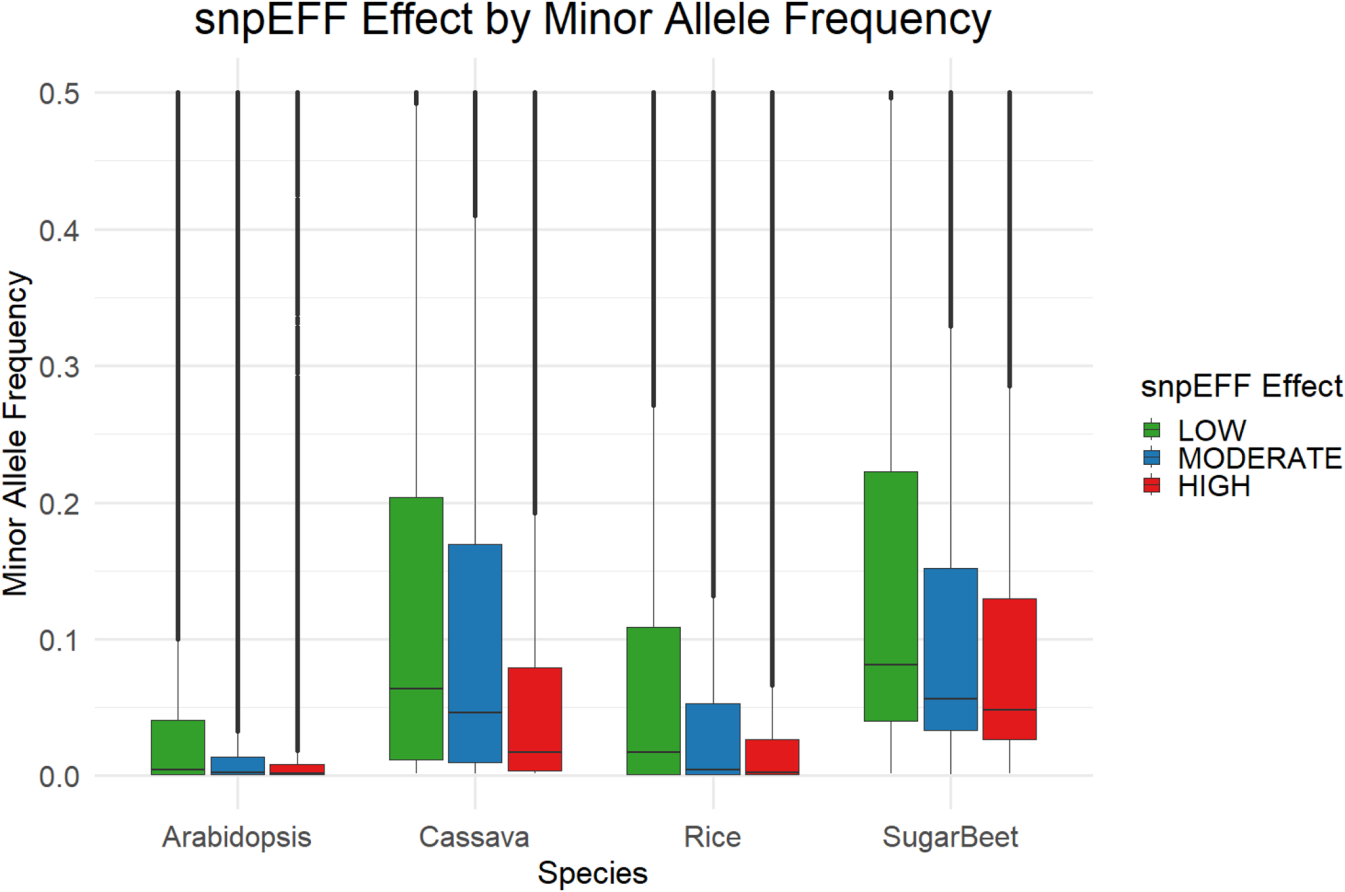
Allele Frequencies Across snpEFF Mutation Classes. Boxplot of relationship between minor allele frequency (MAF) and snpEFF mutation effect. Mutation effects are classified as low (synonymous), moderate (nonsynonymous or in-frame small indels), and high (frameshift, splice site variants, start-codon loss, etc)

Our evaluation of the mutational landscape over 100k genes across four plant species displays how selection pressures are shaped by the functional elements of protein structure. We found that as expected, using snpEFF as our mutation classifier, high effect mutations are far rarer than low or moderate effect mutations as they generally involve small indels or single nucleotide variants in start codons, stop codons, or splice sites (Fig. 2). While moderate effect mutations are expected to have more impact on functional variation, they are statistically more common than low effect mutations due to the number of mutations that change amino acid sequence. We see that moderate effect mutations have a makeup a comparable proportion to low effect mutations in each of the species (Fig. 2). Across the proteins of each plant species (except rice) we observed a relative enrichment in moderate and high effect mutations towards the C-terminus and N-terminus of the protein. However, the abundance of each mutation class, referring to the number of mutation sites along the genome in a population, is not synonymous with the total frequency of these mutations in a population. To accommodate this we analyzed the distribution of these mutation types with and without a 5% MAF filter (Fig. 2,Fig. S1). Moderate and high effect mutations have lower average allele frequencies compared to low effect mutations (Fig. 3). While MAF is generally useful as a measure of selection pressure within a population, the differences in populations structure make cross species comparison difficult, however the trends between mutation effects are still consistent among each plant species.

**Fig. 4:**
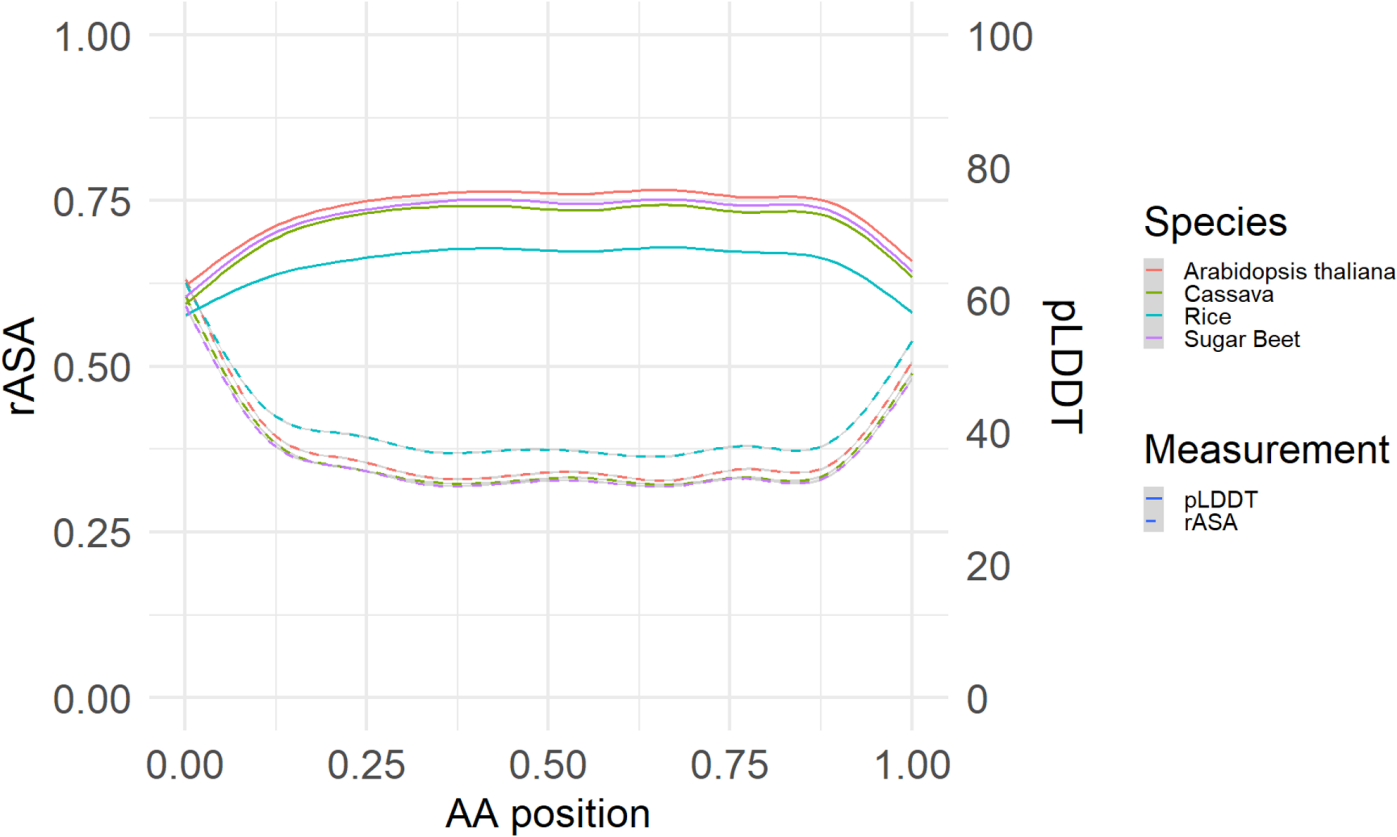
Relationship Between Amino Acid Positions and Protein Structure. The relative available surface area (rASA, left y-axis) and predicted local distance difference test (pLDDT, right y-axis) along the relative position of each amino acid in its respective protein is plotted with the geom_smooth function in ggplot2 using a general additive model with 95% confidence intervals shown as shaded areas. One million amino acid positions were sampled to perform this plot and analysis

To address how mutation characteristics might be related to proteins structure we first investigated the relationship among protein structure properties. We found that the pLDDT folding confidence score is diminished toward the C-terminal and N-terminal regions of the proteins of each plant species (Fig. 4). Conversely the rASA value of the amino acids in these regions was also higher, indicating a propensity towards having an unfolded or disordered structure. Fittingly, rASA and pLDDT are negatively correlated (R=-0.47, *p* < 2.2e-16) indicating protein folding algorithms are more confident in predicting more structured/ordered regions of the protein, and this correlation remained nearly identical even when only considering the core or middle 50% of protein sequences. For Arabidopsis, we also evaluated how these protein characteristics correlated with evolutionary conservation which showed positive correlation with rASA (Fig. S2). The rASA measure of protein disorder also showed negative correlation with IUPred2A protein disorder annotation (Fig. S3).

**Fig. 5:**
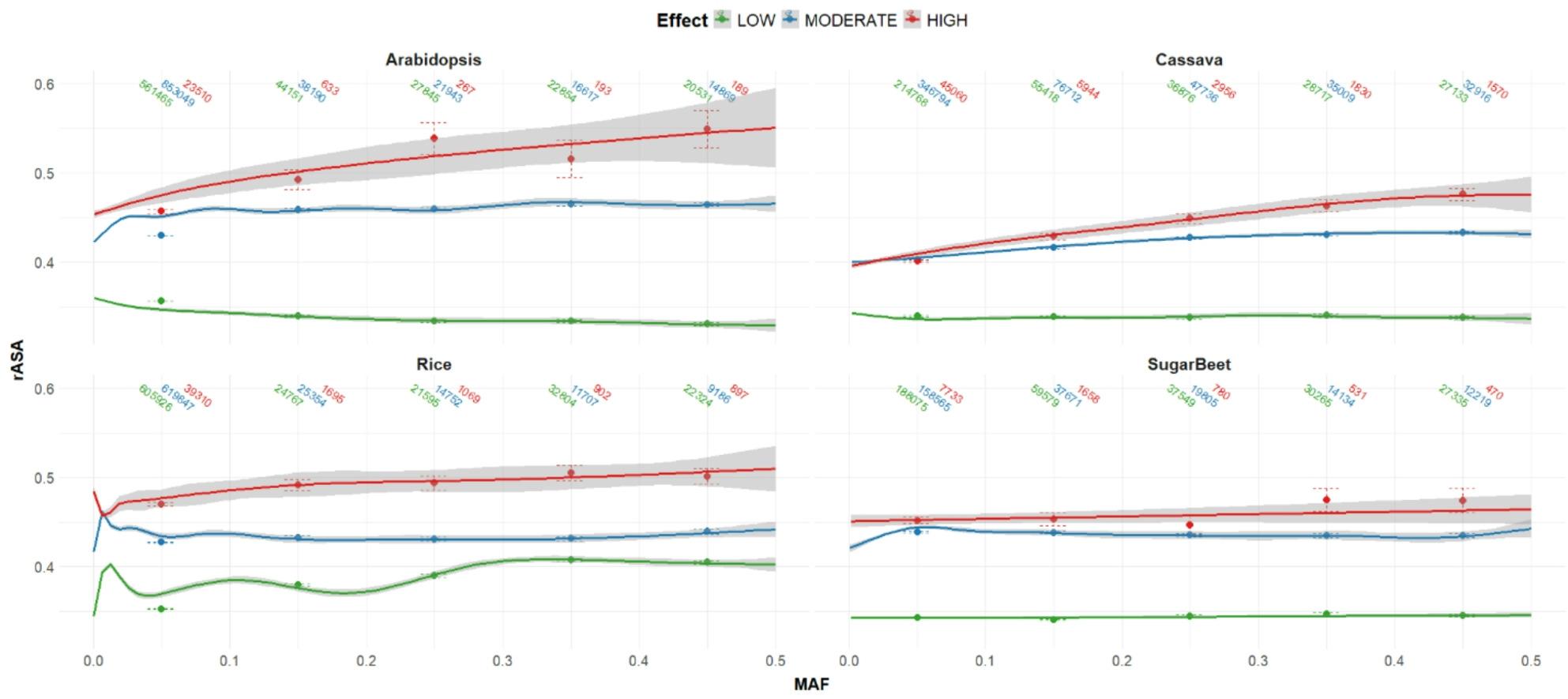
snpEFF Mutation Classes Across Protein Structures. The relationship between minor allele frequency (MAF) and relative available surface area (rASA) at mutation positions in protein coding genes is plotted with the geom_smooth function in ggplot2 using a general additive model with 95% confidence intervals shown as shaded areas. Mutation counts, means, and standard errors binned by minor allele frequencies are displayed. Mutation effects are classified as low (synonymous), moderate (nonsynonymous or in-frame small indels), and high (frameshift, splice site variants, start-codon loss, etc)

Finally, we combined the protein structure annotations with the mutation effects and selection signatures to evaluate their relationships. We found that in all plant species, mutation classes showed a serial relationship with the rASA of their respective AAs, high effect mutations occurring in high rASA residue, low effect mutations in low rASA residues, and moderate effect falling in between (Fig. 5). Pairwise Wilcoxon ratio tests of rASA between effect classes in each species showed significant differences between classes (*p* < 4.2e-07), except for the comparison between moderate and high effect mutations in cassava (*p* = 0.11). This relationship showed some variability when disregarding the beginning and ending 10% tails of the proteins, however high and moderate effect mutations still tend to occur in AAs with higher rASA than low effect mutations (Fig. S4). Overall, the high and moderate effect mutations showed positive, significant correlations between MAF and rASA (*p* < 2e-15), while low effect mutations did not show any consistent relationship (Fig. 5). A likelihood ratio test between a linear mixed model controlling gene identity, amino acid, and amino acid position indicating a significant relationship between MAF and rASA (*p* < 2e-16).

**Fig. 6:**
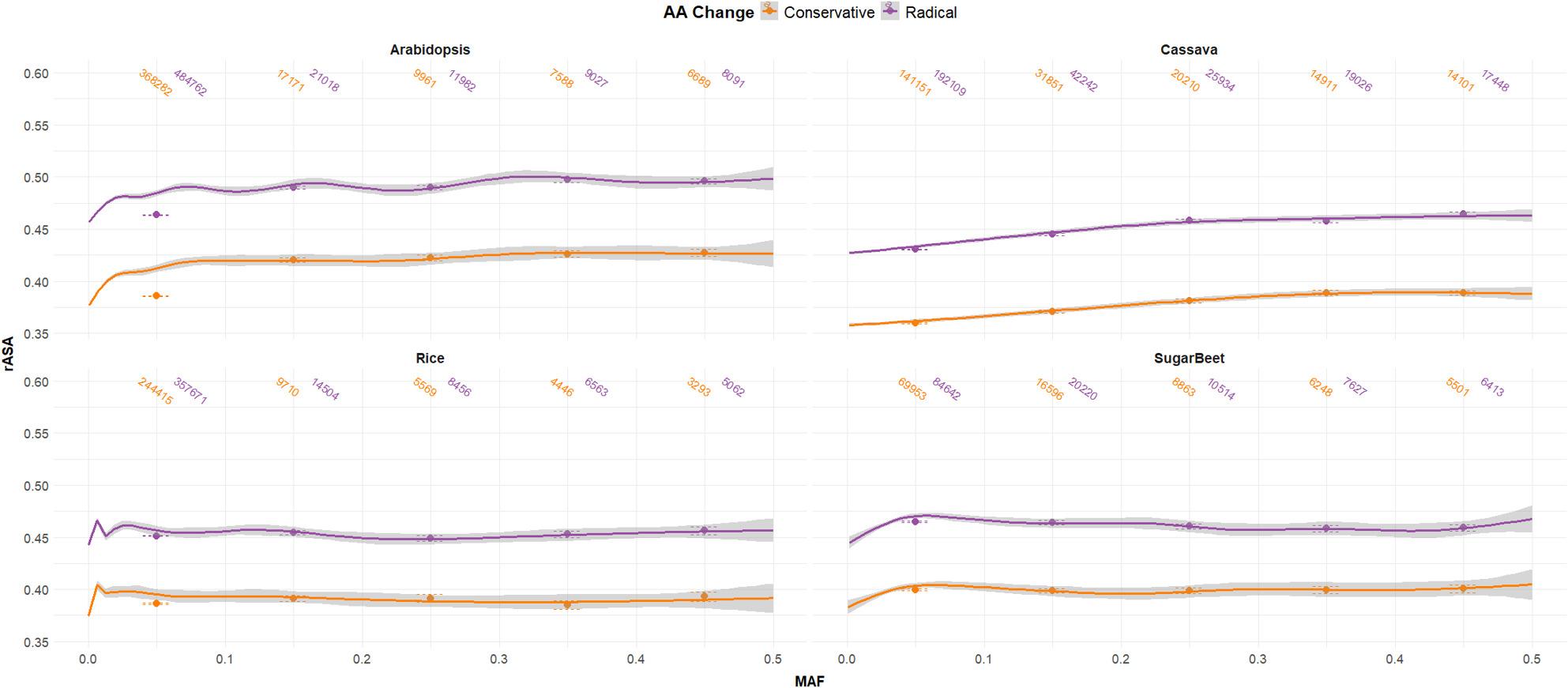
Amino Acid Substitution Across Protein Structures. The relationship between minor allele frequency (MAF) and relative available surface area (rASA) at nonsynonymous mutation positions is plotted with the geom_smooth function in ggplot2 using a general additive model with 95% confidence intervals shown as shaded areas. Mutation counts, means, and standard errors binned by minor allele frequencies are displayed. Nonsynonymous mutations are divided into those which result in an AA in the same functional group (Conservative) and those that result in an AA in a different functional group (Radical)

Additionally, we further investigated each mutation type according to other characteristics. Moderate, nonsynonymous mutations were classified by their resulting amino acid change as conservative (changing to an AA in the same functional group) or radical (changing to an AA in a different functional group). Radical AA change consistently occurred in AA residues with higher rASA in each species, while conservative AA changes showed trends similar to low effect mutations (Fig. 6). For a more nuanced approach we also considered BLOSUM [19] scores of every AA change, but the only clear trend shown was that mutations with BLOSUM score of > = 3 occurred in lower at AA with lower rASA than mutations with lower BLOSUM scores (Fig. S5). Synonymous mutations were evaluated for whether protein structure showed any association with codon bias, but found little evidence (Fig. S6). We broke out high effect mutations by their resulting impact (Fig. S7). This analysis showed stop-gain mutations occurring at very high rASA locations along the protein, consistent with what was observed in C-terminal regions of proteins showing high accessibility/disorder.

## Discussion

In this study we present a pipeline for evaluating mutations regarding their context within protein structures. Additionally, we use this tool to analyze relationships between protein structure and mutations’ effects and frequency across four plant species: Arabidopsis, cassava, rice, and sugar beet.

### Relative amino acid position

While general structures and trends among protein structures are well understood, the consistent trends across the positional characteristics of the amino acids in proteins can shed light on their functional importance. The data showing consistent enrichment for high effect mutations at the C-terminal and N-terminal regions of the protein suggests that there is diminished functional impact of mutations in these regions (Fig. 2). One explanation for this enrichment is the fact that certain classes of high effect mutations (stars-lost, stop-loss) are only possible at the ends of the protein, however this suggests some functional redundancy in the C-terminus of the protein as losing start codons is tolerated. There is evidence in human proteins for a propensity for multiple in-frame start codons, a redundancy that may allow for this enrichment of C-terminal high effect mutation [20].

Proteins’ structures and functions are diverse and complex, however general trends show consistency among proteins and the plant species we’ve evaluated. The C-terminal and N-terminal regions of the proteins tend to have higher rASA indicating that they are less likely to be involved in the structural complex of the protein, but rather exist as loose or unstructured tails (Fig. 4). Our assessment of rASA is supported by its correlation with evolutionary conservation (PhastCons) and IUPred2A disorder annotation in Arabidopsis.

Much research has been done to study the function of disordered protein tails and their function in signaling, binding, and localization [21]. The enrichment of C-terminal disordered regions has been associated with transcript factor activities [22]. Transmembrane proteins can have important disordered regions including hydrophobic tails necessary for situating within a membrane [23], and similarly holds true for important functions in channel proteins [24]. N-terminal disordered regions have been shown to be important for DNA binding and scanning [25].

### Selection pressure on protein structure

The population allele frequencies across all protein structures shed light on patterns of selection and different factors in functional importance. The minor allele frequency in a large population can serve as a rough metric for selection pressure occurring at a locus [26, 27]. Low minor allele frequencies indicate rare and potentially deleterious mutations, while high minor allele frequencies can suggest a more neutral selection pressure at the locus. While this is only a general trend, and does not account for genetic drift and young mutations, it is supported by the annotated mutation effects (Fig. 3). Across all species, high effect mutations had low frequencies, low effect mutations had high frequencies, and moderate mutations fell somewhere between the two extremes.

We found that there is a clear relationship between the effect of a mutation and the relative position in a 3D protein in which it is likely to occur (Fig. 5). The high effect mutations were more likely to be found at AA positions with higher rASA compared to other mutation effects. Similarly, moderate effect mutations were found at higher rASA positions than low effect mutations. Within the nonsynonymous moderate effect mutations, the AA substitutions that changed the functional class of the AA (radical) also occurred at higher rASA positions than those that did not change the functional class (conservative, Fig. 6). Interestingly, a slight positive correlation between rASA and MAF is seen among high effect mutations (Fig. 6), which suggests that less negative selection pressure may be exerting on these regions of the protein. It is worth noting that most statistical tests performed in this study were highly significant, however a large component of this significance is due to the large sample space being evaluated (many hundred thousand mutations in each species). It is therefore very important to also consider magnitudes and relative impacts of correlations and associations, and how much variation is explained be each cofactor.

### Species heterogeneity

The overall trends and relationships between protein structure and selection pressure presented here are consistent among the plant species we’ve examined, however there are sizeable differences exist between the plant species’ mutation landscapes. Cassava and sugar beet are naturally outcrossing species, with cassava also heavily relying on clonal propagation, and this may explain the generally higher minor allele frequencies, especially among high effect mutations that may be masked by heterozygosity (Fig. 3). The varied sources of each of the species’ datasets including population size, population structure, sequencing and genotyping pipeline make any cross-species comparisons inherently speculative. The consistent patterns of protein structure among the species, however, are reassuring given this large heterogeneity.

### Utility and future directions

Understanding the importance of protein structure and its relationship to selection and function can help improve models predicting mutation effects. Previous papers have used evolutionary conservation and protein features to estimate deleterious mutations to dissect their impact on genomic selection, plant breeding, and plant improvement [28–30] and these types of models may be improved by directly incorporating predicted 3D structures previously unavailable. The success of recent deep learning methods such as PlantCaduceus [31] or ESM [32] predicting mutations effects, trained directly on DNA sequence, suggests the protein effects are already trackable directly from large datasets of DNA sequence, however our results here may help interpret the underlying attributes of these predicted effect. In its simplistic application, relating protein structure to a mutation may be used as a secondary axis of variation to rank putative causative mutations among possible candidates [33, 34].

One possible oversight in considering point mutations and their effect on protein structure is the omission of mutational context. Individuals in a species may contain mutations at multiple positions compared to the reference or ancestral gene used to generate the default protein structure. An alternative method to measure the protein function would be to generate 3D structures for each mutated protein in a population and use 3D structure alignment tools such as Foldseek and TM-Align [35, 36] to compare structural differences imposed by mutations. This may offer a way to detect novel functional states otherwise undetected, though the ability for protein folding algorithms to act at this scale of precision is a currently debated topic [4, 5].

## Conclusion

In this study, we developed a comprehensive pipeline for evaluating mutations within the context of protein structures and applied this tool to analyze the relationship between protein structure, mutation effects, and their frequency across four plant species: Arabidopsis, cassava, rice, and sugar beet. Our findings highlight several key insights into the positional characteristics of amino acids and their functional importance within protein structures. Our study underscores the importance of considering the positional context of amino acids within protein structures when evaluating mutation effects. The consistent trends observed across diverse plant species provide valuable insights into the functional implications of mutations and the evolutionary pressures shaping protein structures. As we generate models that are used to evaluate, or even design, mutations and predict their effects on protein function it will be valuable to consider the relative impact on the structure of the protein.

## Supplementary Information

Supplementary Material 1.

## Data Availability

Code used to produce results can be found in the github repository corresponding to this manuscript: https:/github.com/em255/PopulationPDBStats. Intermediate files can be downloaded from Zenodo repository - 10.5281/zenodo.17203176.
