# Supplementary material for: Protein structure and selection pressure in plants: using mutation to understand the functional importance of protein structure
Source: BMC Genomics. 2026 Feb 24;27:328. doi: 10.1186/s12864-026-12674-2 (PMC13037108; doi:10.1186/s12864-026-12674-2)
Supplement: Supplementary file 1 — Supplementary Material 1. [file 12864_2026_12674_MOESM1_ESM.zip › SupplementalFiguresCombined.pdf]

Supplementary Figures

Protein Structure and Selection Pressure in Plants: Using Mutation to Understand the Functional Importance of Protein Structure

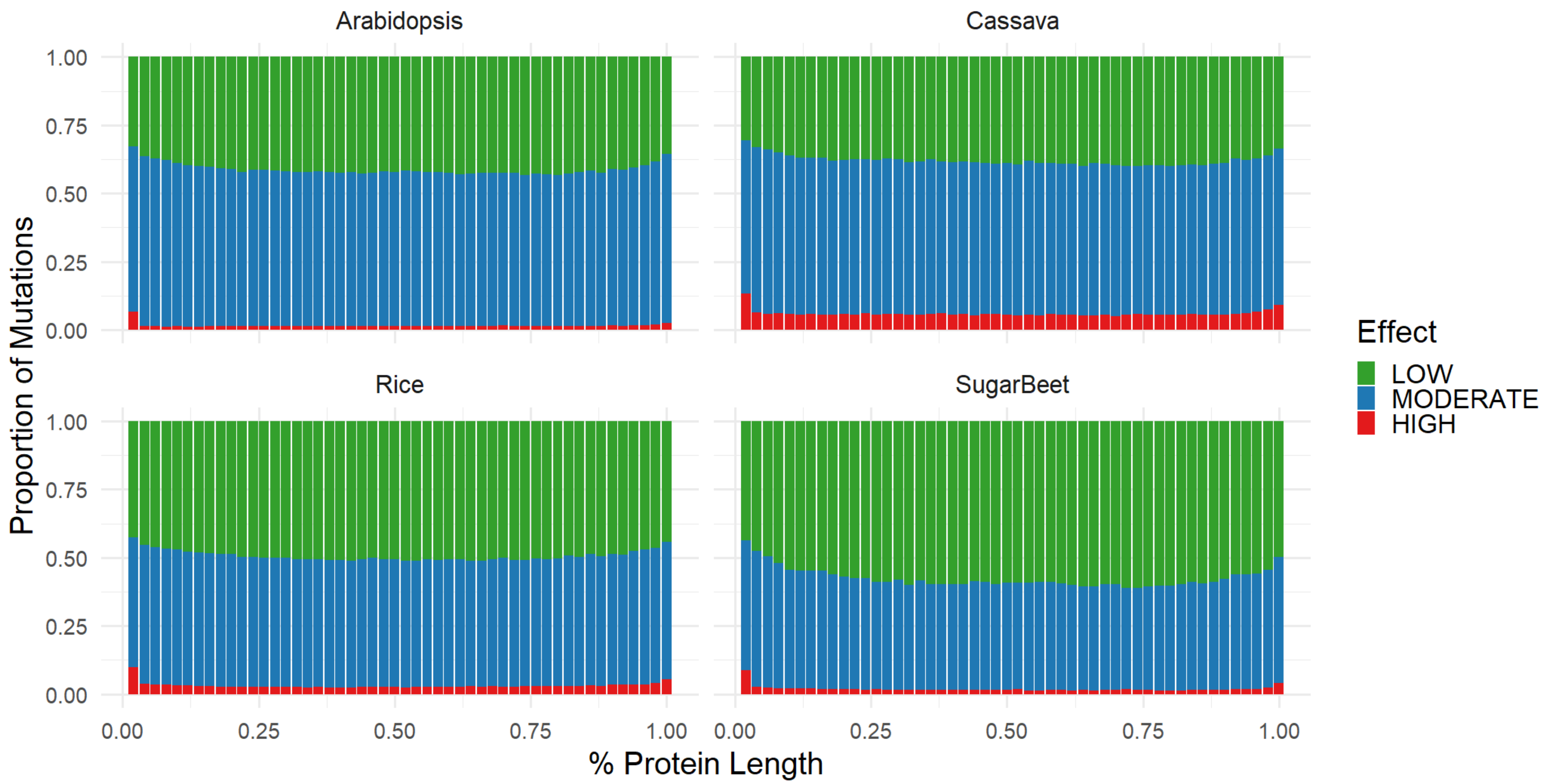

Figure S1. Relationship Between Amino Acid Positions and snpEFF Mutation Classes. The proportion of mutations that fall into each snpEFF mutation class is plotted along the relative position of each amino acid of every protein coding gene in each plant genome. Mutation effects are classified as low (synonymous), moderate (nonsynonymous or in-frame small indels), and high (frameshift, splice site variants, start-codon loss, etc).

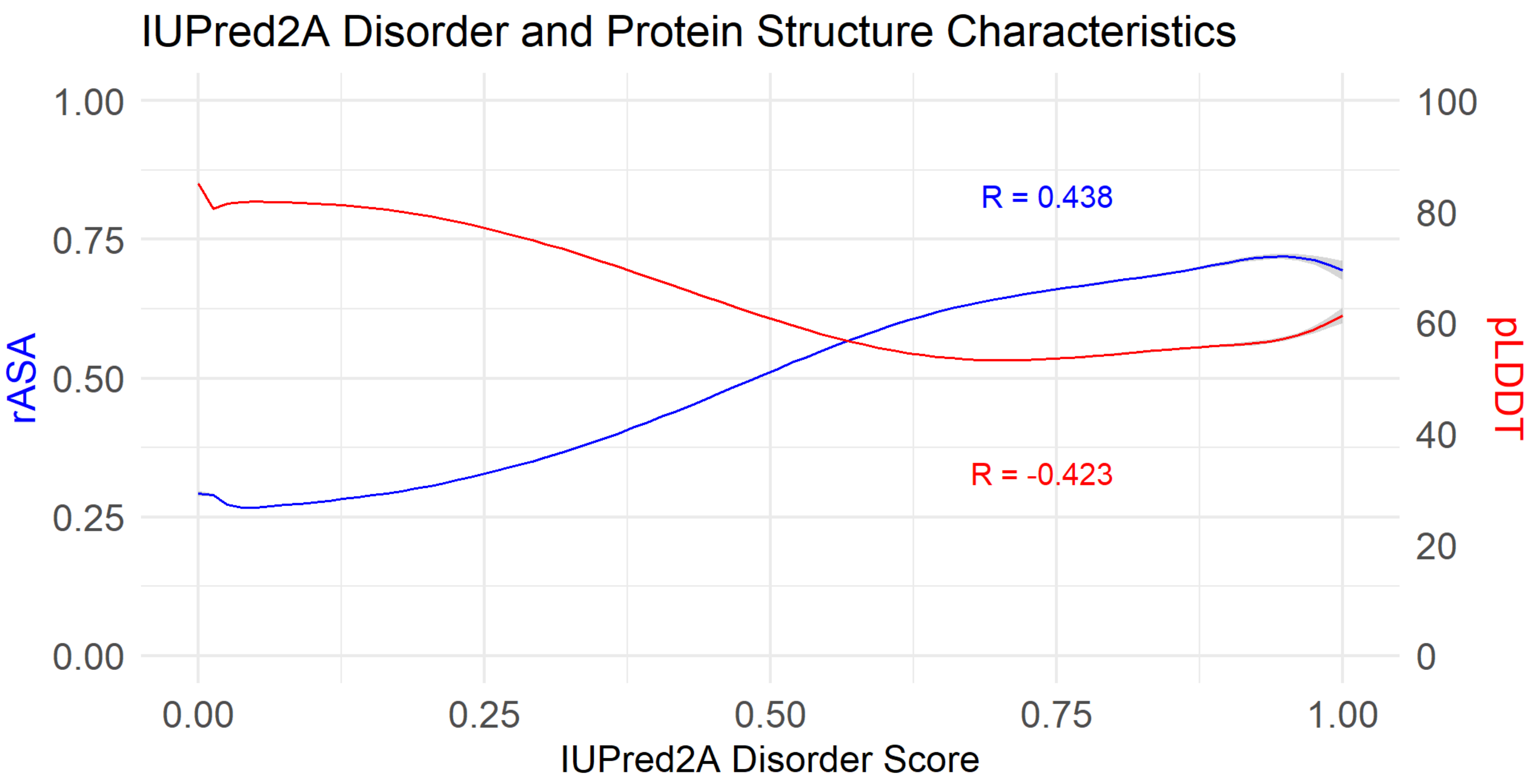

Figure S2. Relationship Between IUPred2A Disorder Score and Protein Structure. The relative available surface area (rASA, left y-axis) and predicted local distance difference test (pLDDT, right y-axis) for 1M sampled protein positions in Arabidopsis are plotted against IUPred2A with the *geom_smooth* function in ggplot2 using a general additive model with 95% confidence intervals shown as shaded areas.

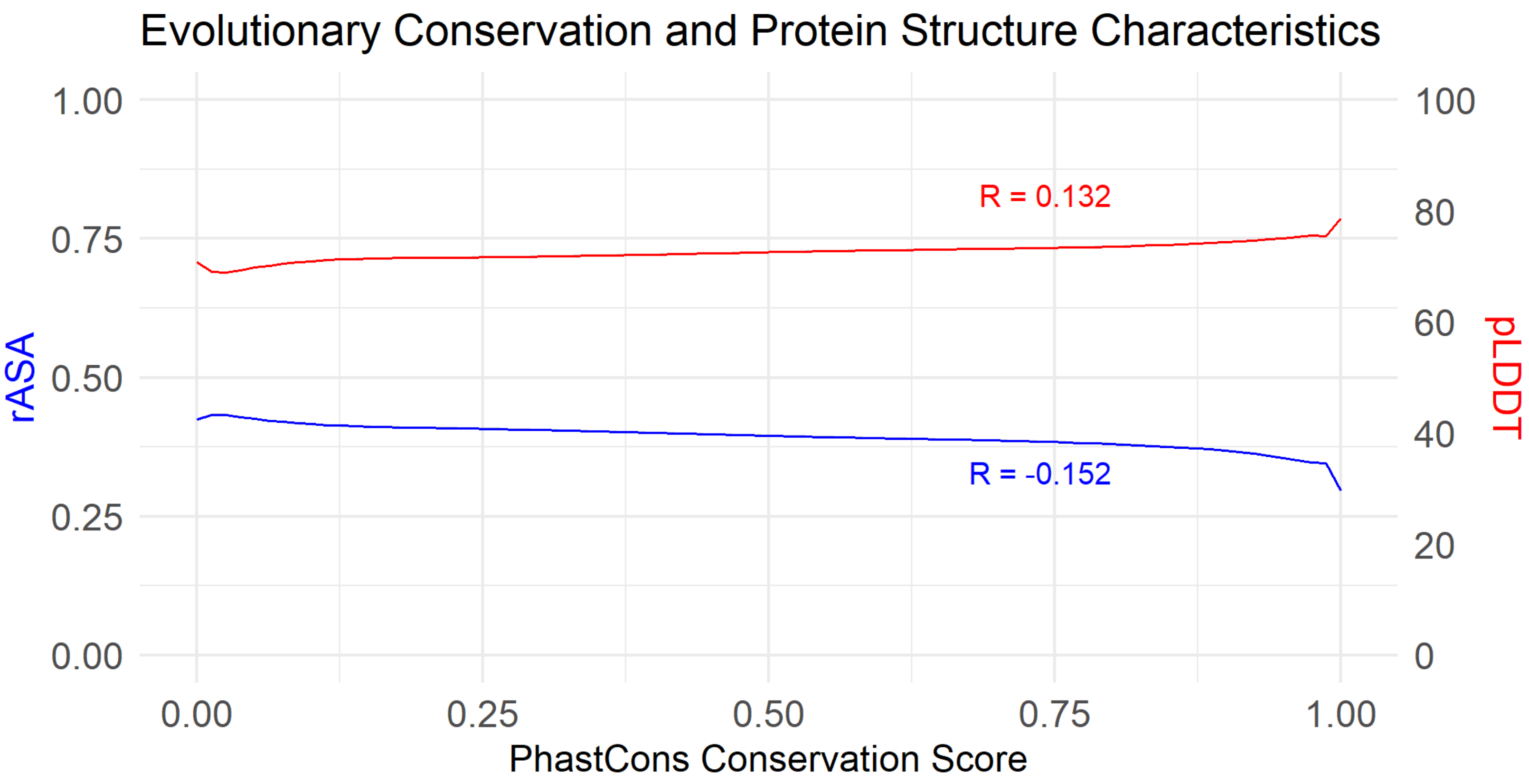

Figure S3. Relationship Between PhastCons Evolutionary Score and Protein Structure. The relative available surface area (rASA, left y-axis) and predicted local distance difference test (pLDDT, right y-axis) for 1M sampled protein positions in Arabidopsis are plotted against IUPred2A with the *geom_smooth* function in ggplot2 using a general additive model with 95% confidence intervals shown as shaded areas

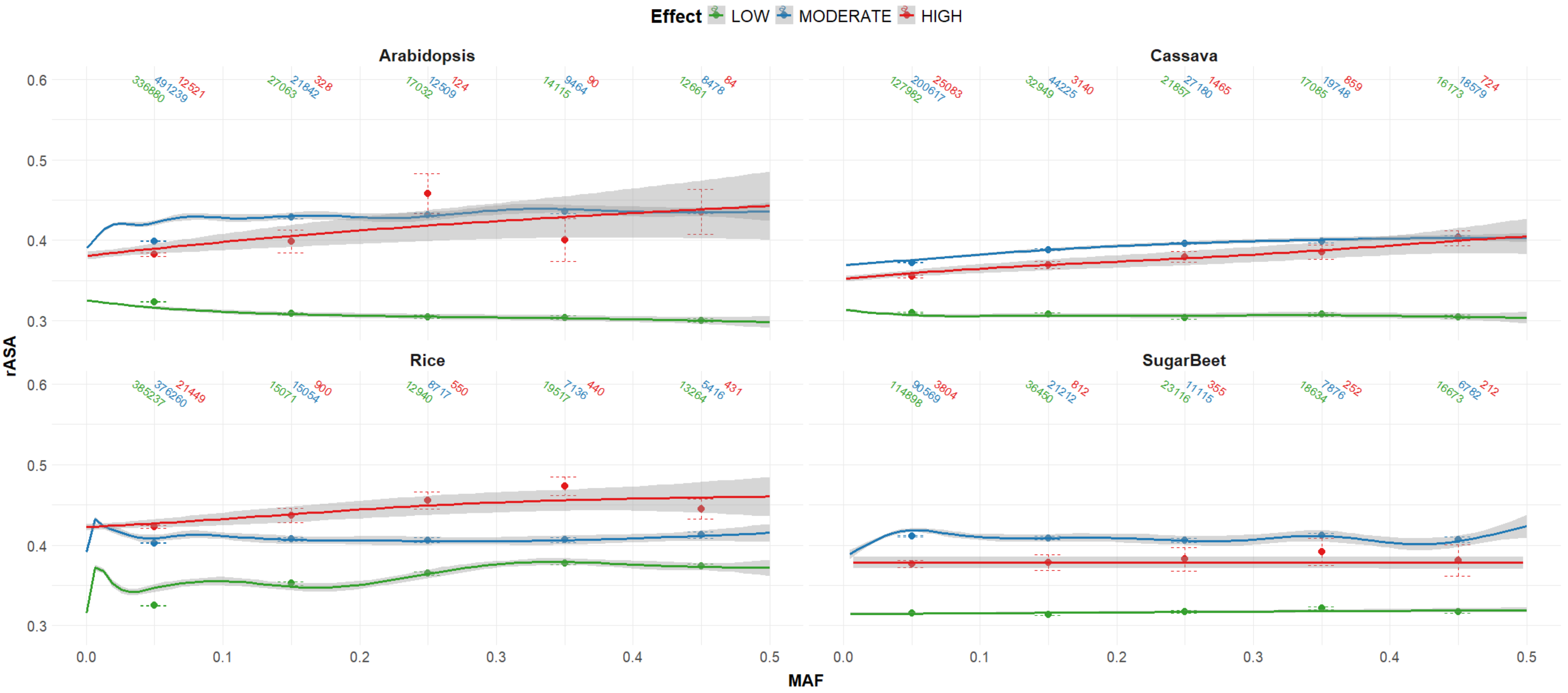

Figure S4. snpEFF Mutation Classes Across Protein Structures (10% tails excluded). The relationship between minor allele frequency (MAF) and relative available surface area (rASA) at mutation positions in protein coding genes is plotted with the *geom_smooth* function in ggplot2 using a general additive model with 95% confidence intervals shown as shaded areas. Mutation counts, means, and standard errors binned by minor allele frequencies are displayed. Mutation effects are classified as low (synonymous), moderate (nonsynonymous or in-frame small indels), and high (frameshift, splice site variants, start-codon loss, etc). Amino acids that fell into the first and last 5% of the protein sequence were excluded.

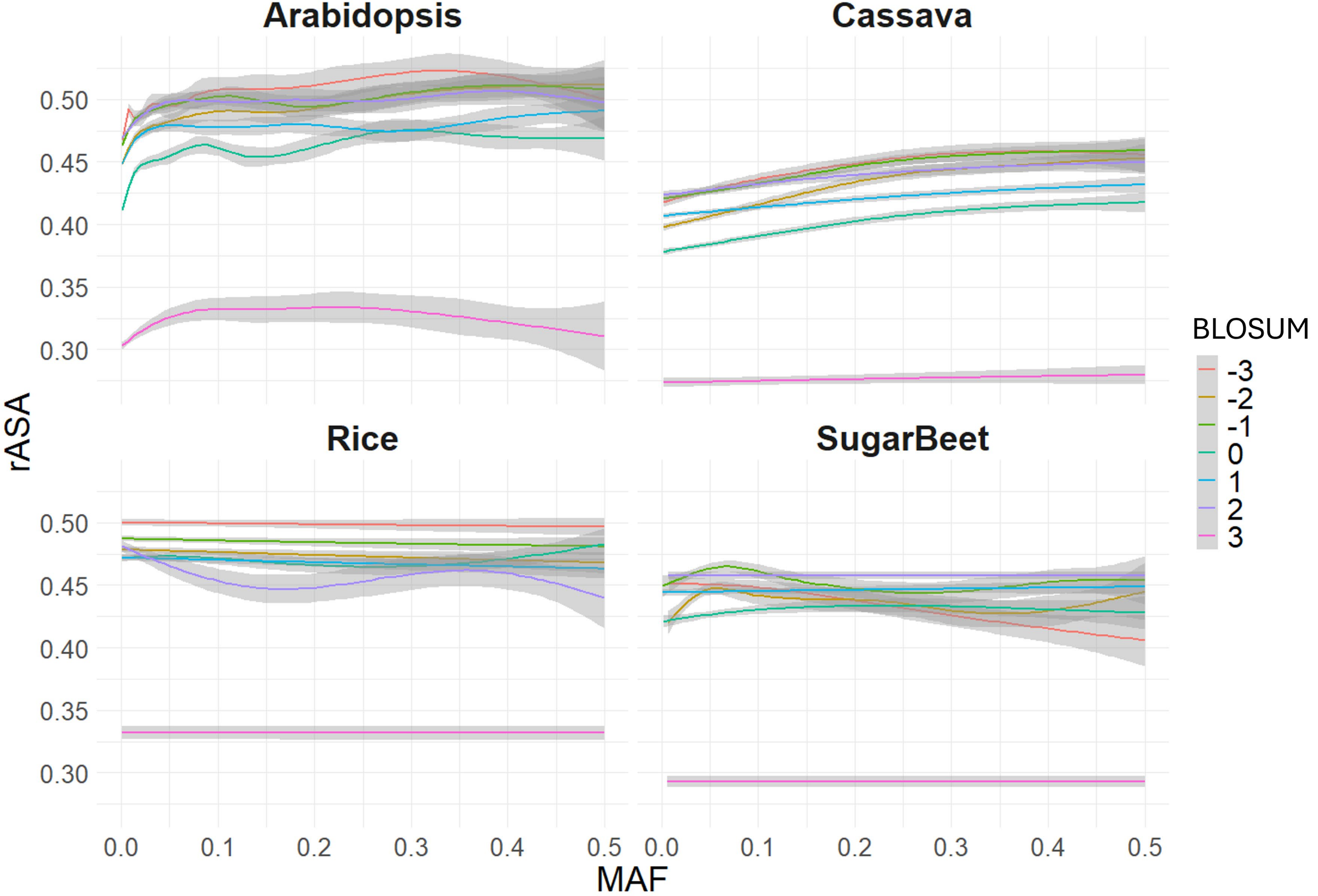

Figure S5. Amino Acid Substitution BLOSUM Scores Across Protein Structures. The relationship between minor allele frequency (MAF) and relative available surface area (rASA) at nonsynonymous mutation positions is plotted with the *geom_smooth* function in ggplot2 using a general additive model with 95% confidence intervals shown as shaded areas. Mutation counts, means, and standard errors binned by minor allele frequencies are displayed. The BLOSUM score given by the BLOSUM62 matrix are shown for nonsynonymous mutations.

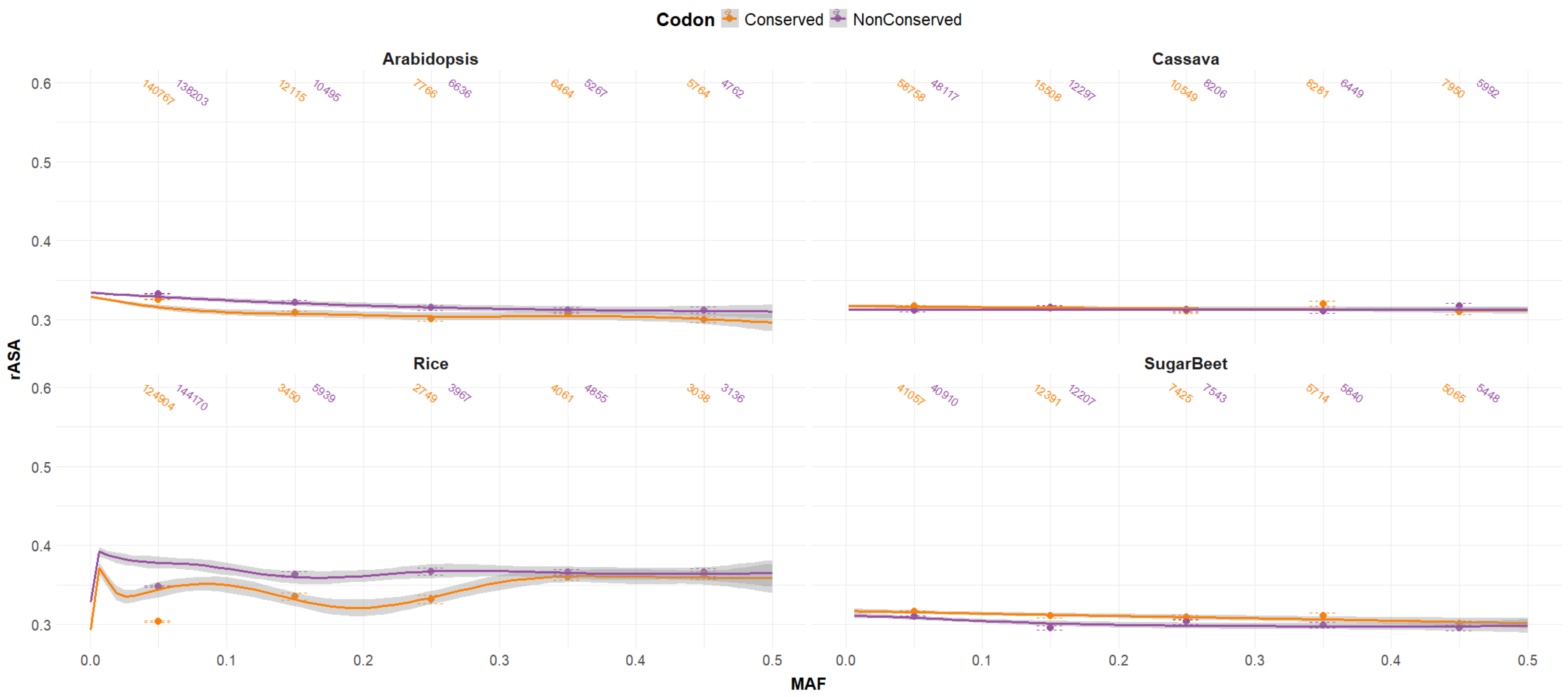

Figure S6. Codon Mutation Class Across Protein Structures. The relationship between minor allele frequency (MAF) and relative available surface area (rASA) at synonymous mutation positions is plotted with the *geom_smooth* function in ggplot2 using a general additive model with 95% confidence intervals shown as shaded areas. Mutation counts, means, and standard errors binned by minor allele frequencies are displayed. Synonymous mutations are divided into those which result in common codons for the amino acid in that species (Conserved) and uncommon codons for the amino acid in that species (NonConserved)

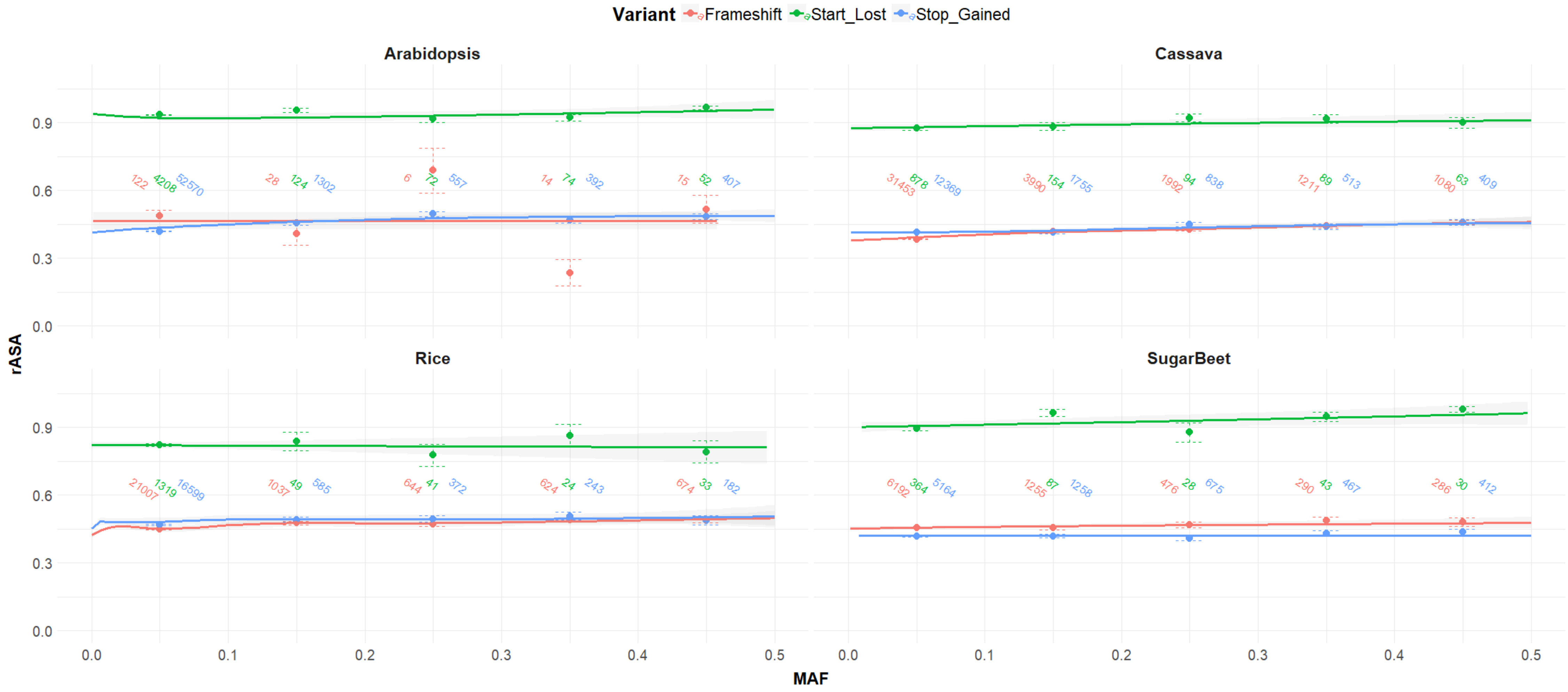

Figure S7. High Effect Mutation Categories Across Protein Structures. The relationship between minor allele frequency (MAF) and relative available surface area (rASA) at high effect mutation positions is plotted with the *geom_smooth* function in ggplot2 using a general additive model with 95% confidence intervals shown as shaded areas. Mutation counts, means, and standard errors binned by minor allele frequencies are displayed. High effect mutations are divided into those that are attributed as frameshift mutations, stop-gained mutations, and start-lost mutations.
