# Supplementary figures and images for: Protein structure and selection pressure in plants: using mutation to understand the functional importance of protein structure
Source: BMC Genomics. 2026 Feb 24;27:328. doi: 10.1186/s12864-026-12674-2 (PMC13037108; doi:10.1186/s12864-026-12674-2)

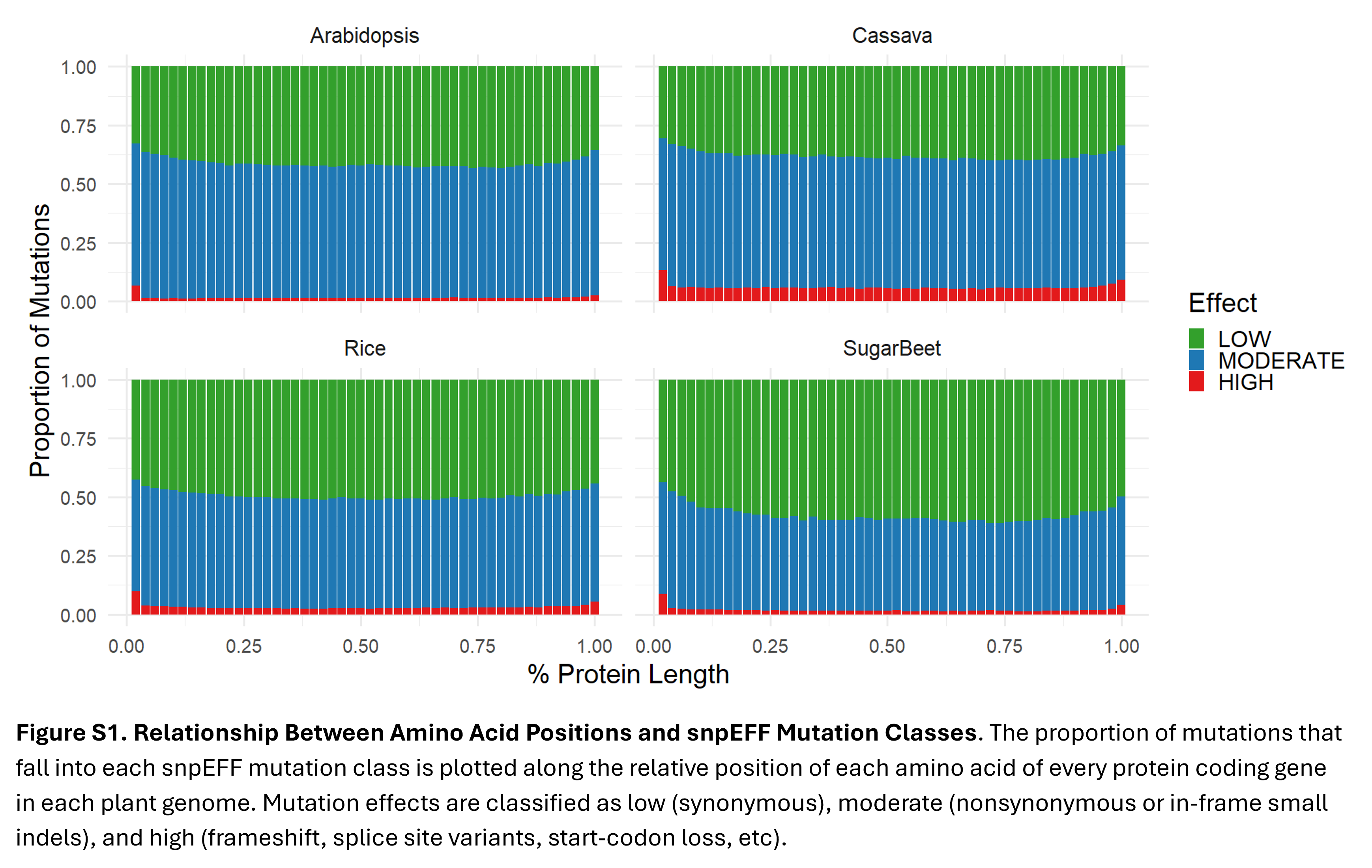

Supplement: Supplementary file 1 — Supplementary Material 1. [file 12864_2026_12674_MOESM1_ESM.zip › FigS1_wthLegend.png]

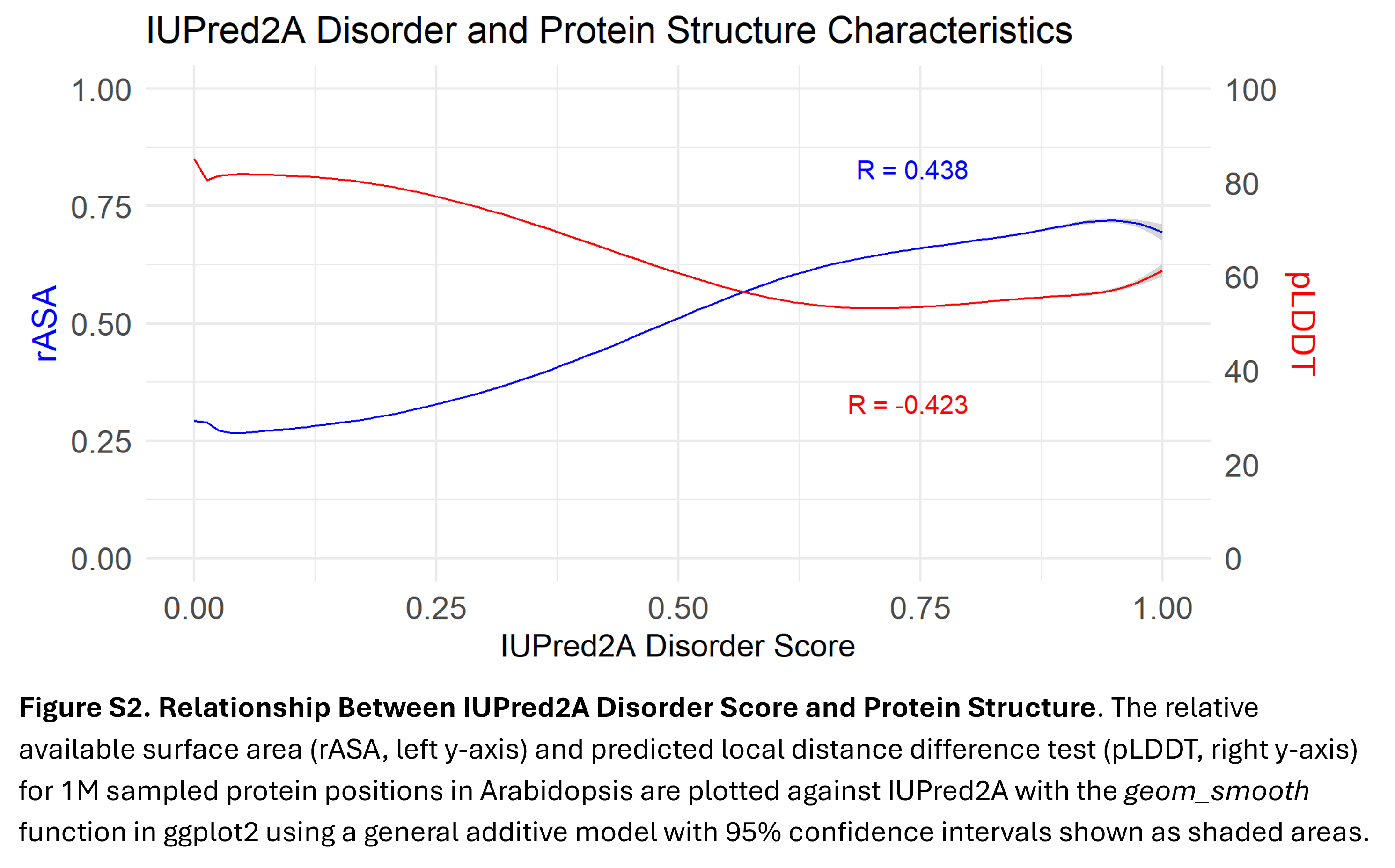

Supplement: Supplementary file 1 — Supplementary Material 1. [file 12864_2026_12674_MOESM1_ESM.zip › FigS2_wthLegend.png]

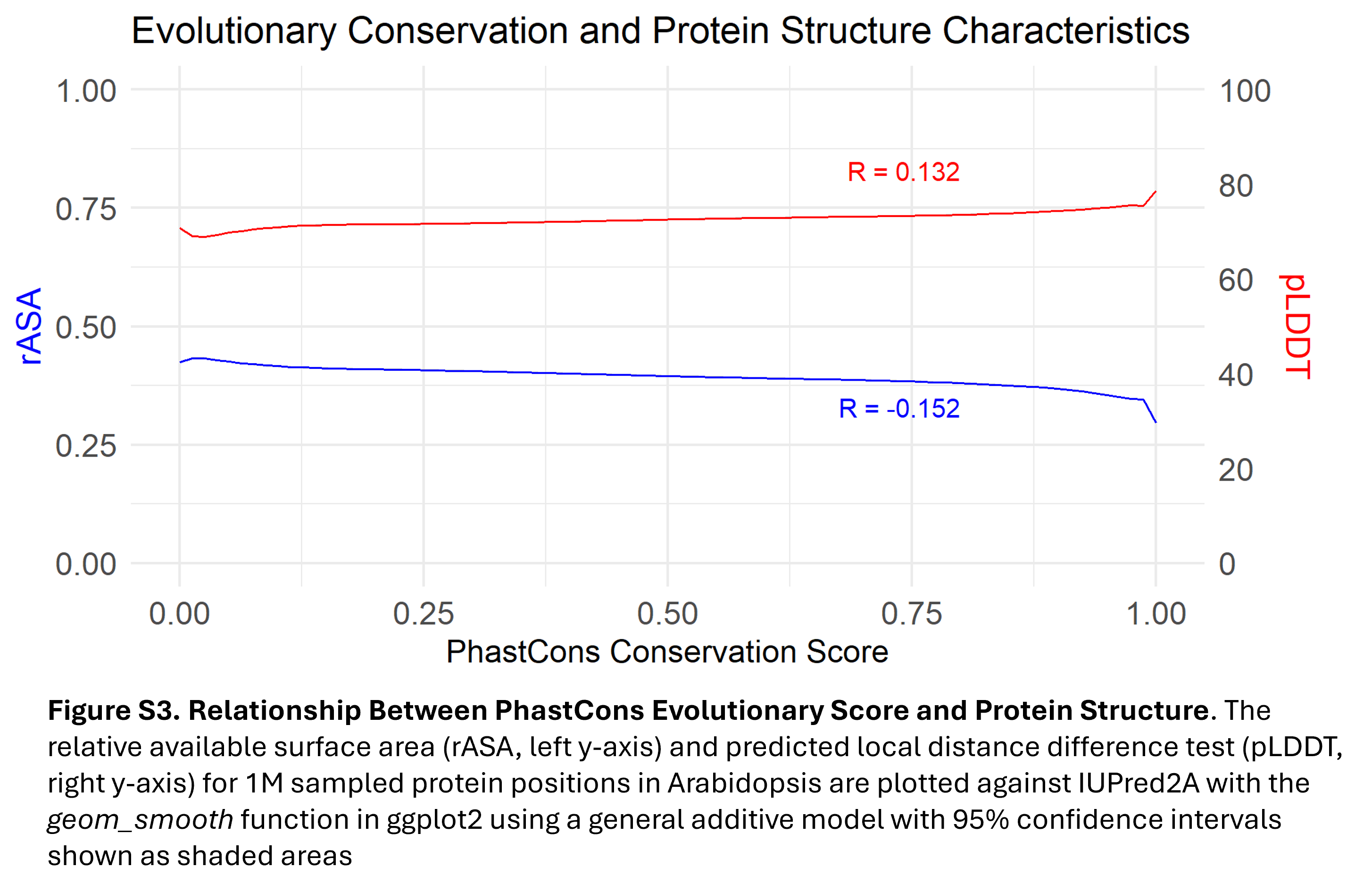

Supplement: Supplementary file 1 — Supplementary Material 1. [file 12864_2026_12674_MOESM1_ESM.zip › FigS3_wthLegend.png]

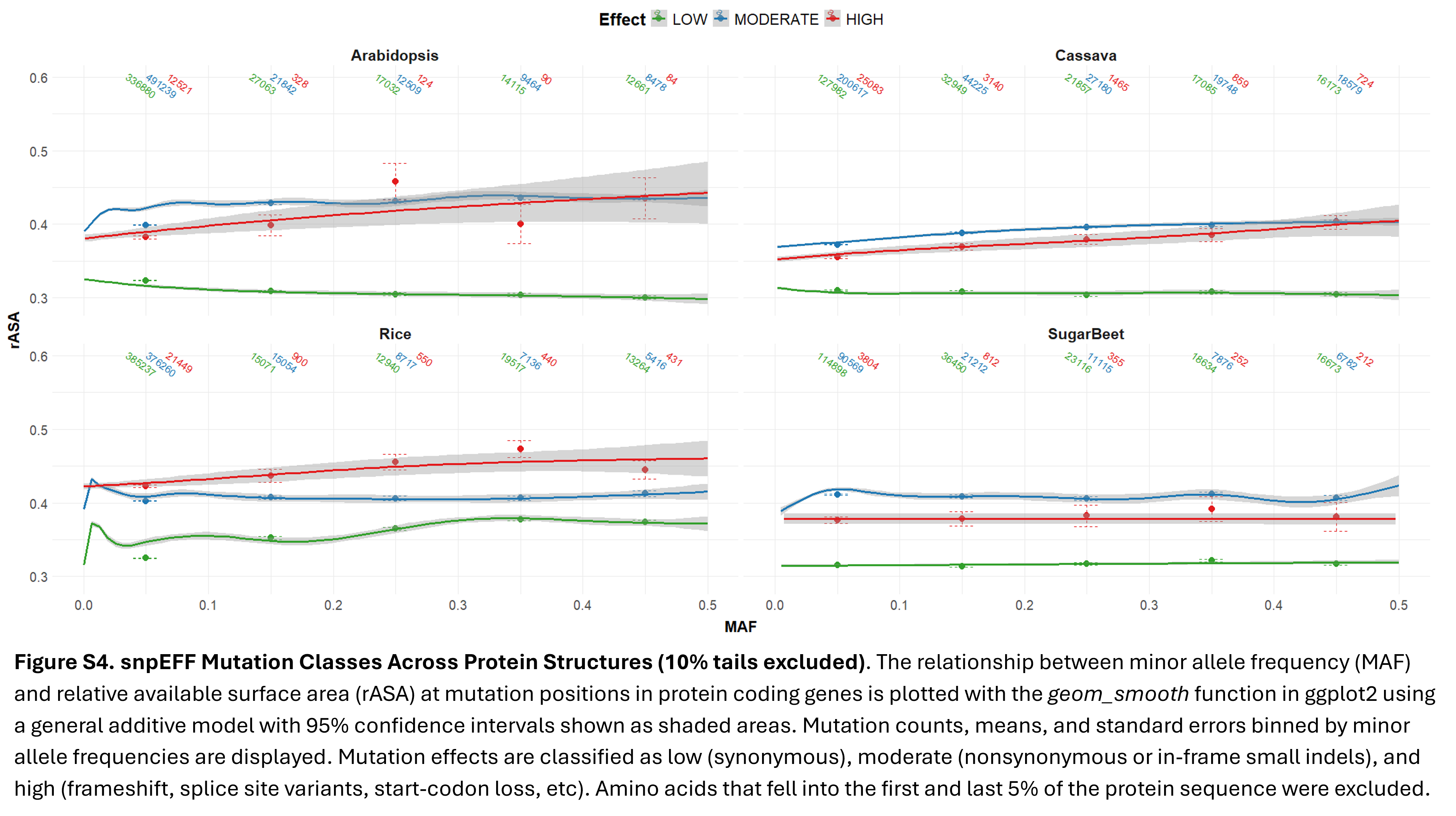

Supplement: Supplementary file 1 — Supplementary Material 1. [file 12864_2026_12674_MOESM1_ESM.zip › FigS4_wthLegend.png]

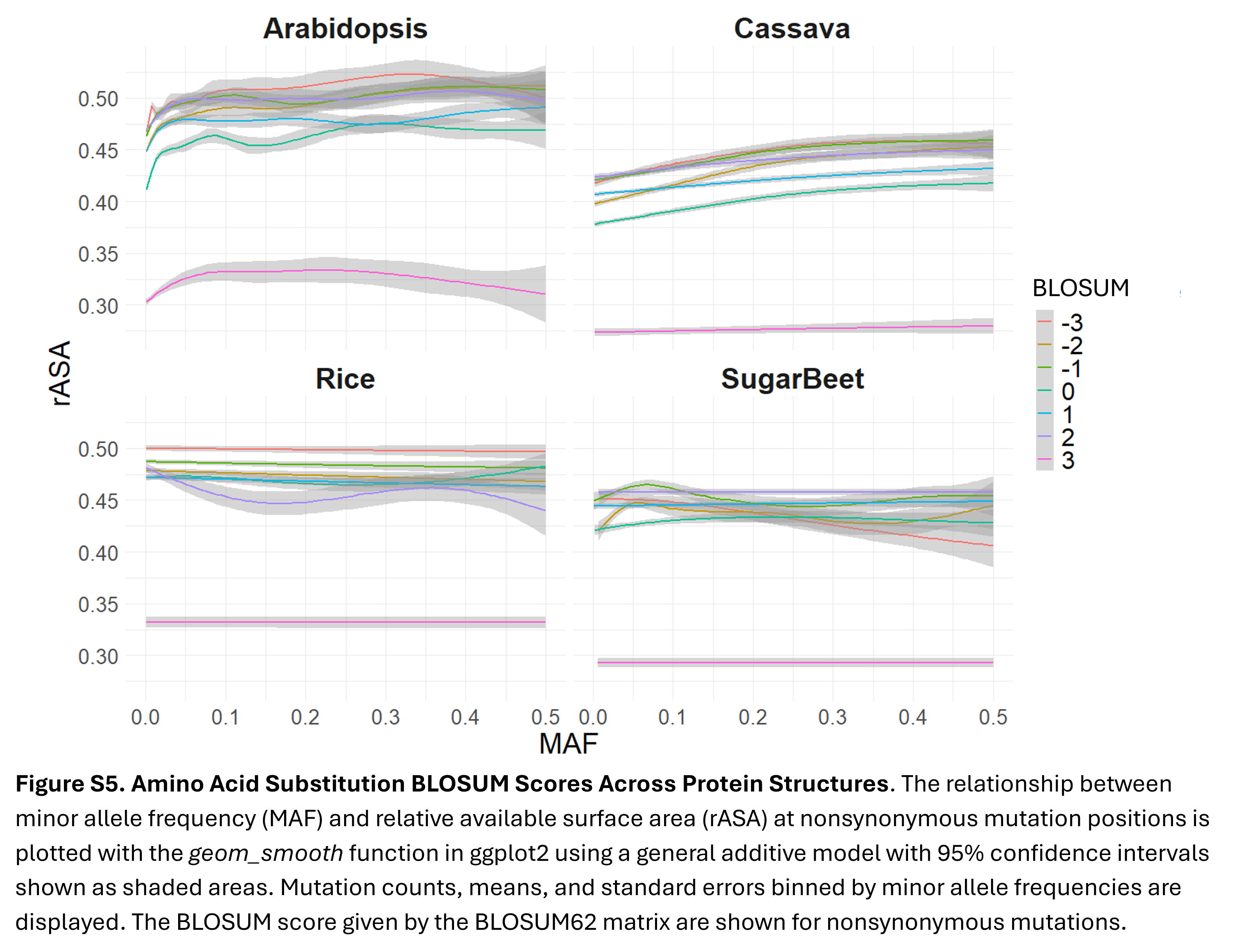

Supplement: Supplementary file 1 — Supplementary Material 1. [file 12864_2026_12674_MOESM1_ESM.zip › FigS5_wthLegend.png]

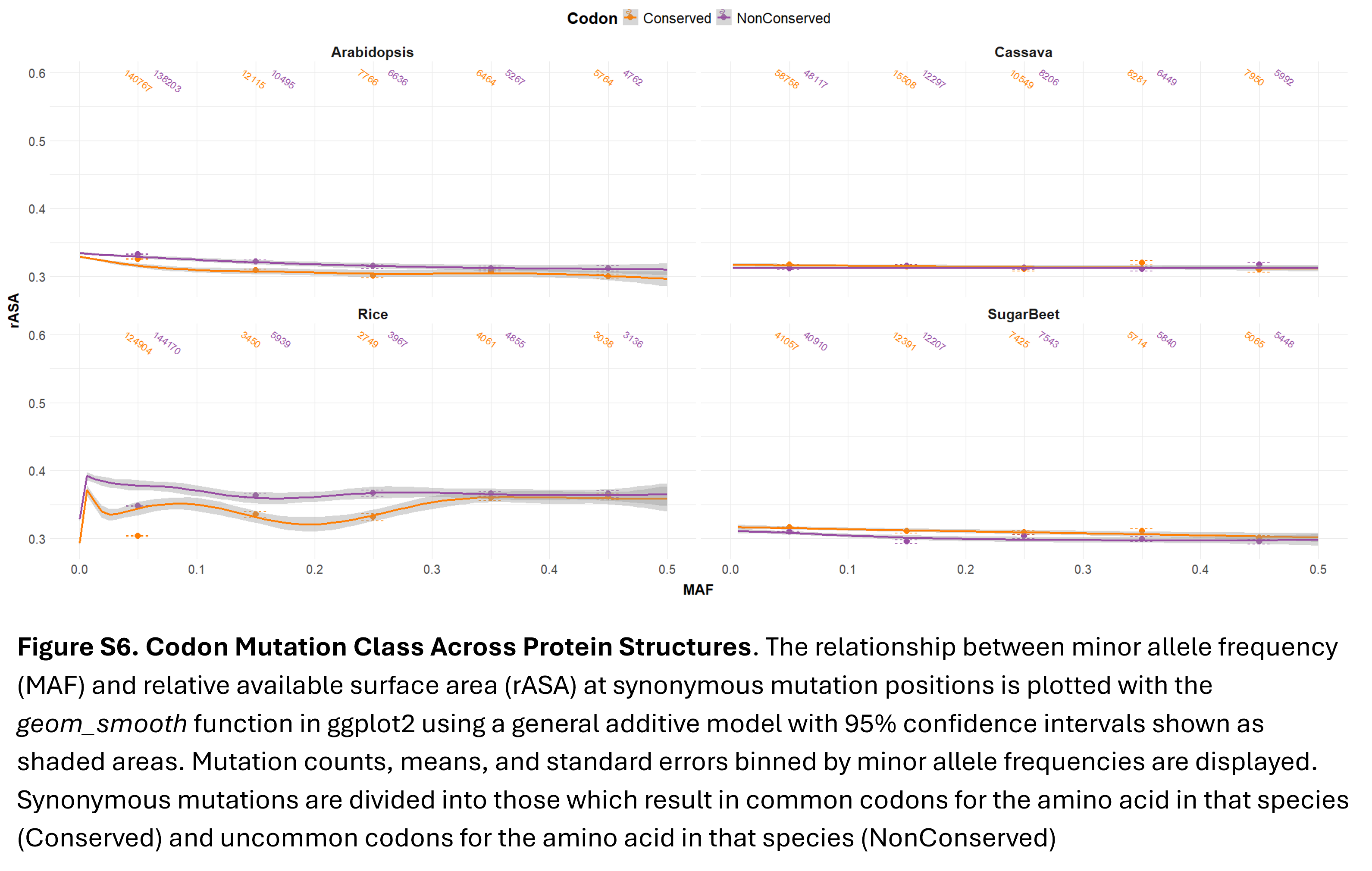

Supplement: Supplementary file 1 — Supplementary Material 1. [file 12864_2026_12674_MOESM1_ESM.zip › FigS6_wthLegend.png]

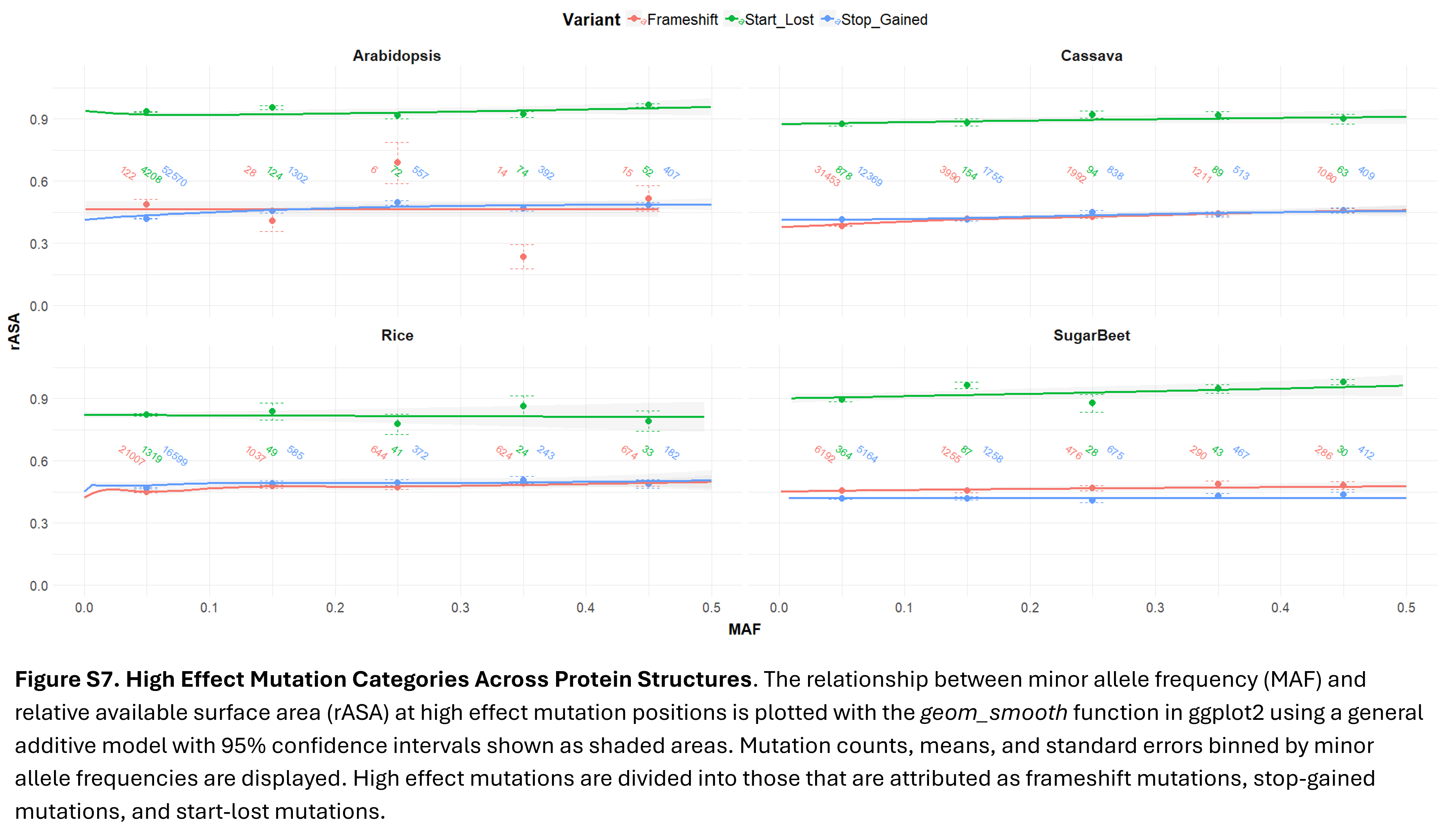

Supplement: Supplementary file 1 — Supplementary Material 1. [file 12864_2026_12674_MOESM1_ESM.zip › FigS7_wthLegend.png]
